# Empirical Bayes for Group (DCM) Studies: A Reproducibility Study

**DOI:** 10.3389/fnhum.2015.00670

**Published:** 2015-12-22

**Authors:** Vladimir Litvak, Marta Garrido, Peter Zeidman, Karl Friston

**Affiliations:** ^1^The Wellcome Trust Centre for Neuroimaging, University College London, Institute of NeurologyLondon, UK; ^2^Queensland Brain Institute and Centre for Advanced Imaging, The University of QueenslandBrisbane, QLD, Australia

**Keywords:** empirical Bayes, random effects, fixed effects, dynamic causal modelling, Bayesian model reduction, reproducibility

## Abstract

This technical note addresses some key reproducibility issues in the dynamic causal modelling of group studies of event related potentials. Specifically, we address the reproducibility of Bayesian model comparison (and inferences about model parameters) from three important perspectives namely: (i) reproducibility with independent data (obtained by averaging over odd and even trials); (ii) reproducibility over formally distinct models (namely, classic ERP and canonical microcircuit or CMC models); and (iii) reproducibility over inversion schemes (inversion of the grand average and estimation of group effects using empirical Bayes). Our hope was to illustrate the degree of reproducibility one can expect from DCM when analysing different data, under different models with different analyses.

## Introduction

Reproducibility is a key issue in cognitive science. It is also an important consideration in the challenging field of dynamic causal modelling (DCM). DCM refers to the inversion and Bayesian model comparison of state space models generating measured timeseries (Friston et al., 2003; Stephan and Roebroeck, 2012). An important and increasingly prevalent application of DCM is to electrophysiological signals, particularly event related potentials as measured with electroencephalographic (EEG; David et al., 2006; Garrido et al., 2007b). Dynamic causal modelling of these data enables experimenters to test hypotheses about causal interactions (or “effective connectivity”) between brain regions, but this is challenging because the underlying generative or forward models are universally ill-posed and nonlinear. Currently, DCMs are inverted by fitting each model individually, using a gradient ascent on variational free energy, which approximates the log model evidence (Friston et al., 2007). This variational Laplace scheme is efficient when the free energy function is well-behaved and has a single (global) maximum (Frässle et al., 2015; Lomakina et al., 2015). However, for more complex DCMs (of the sort used for electrophysiological data) the free energy landscape can be more complicated, with the potential for multiple local extrema. Furthermore, models with different numbers of free parameters differ in the flexibility they afford the inversion scheme, when invading local maxima. The resulting behavior of the inversion scheme might therefore be capricious, leading to poor reproducibility. This is particularly problematic for model comparisons, where the value of the free energy at a maximum plays a crucial role as a proxy for model evidence.

*Post hoc* inference (Friston and Penny, 2011) or Bayesian Model Reduction (BMR) is a promising framework for finessing some of these problems. In this framework, only one model per dataset is fitted using a conventional (e.g., variational Laplace) scheme. This is the full model, from which any other (reduced) model can be derived by removing a subset of its parameters with precise shrinkage priors (i.e., by fixing these parameters to have a prior mean of zero with high precision). The free energy and parameter estimates for these reduced models can be approximated from those of the full model analytically, given the prior of the reduced model. This approximation is exact in the case of linear models. Intuitively, the information that enables BMR comes from the curvature of the free energy function of all parameters at the maximum of the full model. In the linear case, there is only one maximum that, when combined with its curvature, fully specifies the quadratic free energy functions of all reduced models. For functional magnetic resonance imaging (fMRI), BMR has been shown to give results that are similar to the conventional approach, at a fraction of computational cost (Rosa et al., 2012). Recently BMR has been extended to electrophysiological models (Friston et al., 2015a,b) and initial results on synthetic data suggest that it recovers the known ground truth better than the conventional approach of inverting each model separately (Friston et al., 2015a). This counterintuitive improvement, when using BMR, is thought to be due to an increased robustness to local minima and associated convergence problems.

The computational efficiency of BMR makes it possible to implement a hierarchical or empirical Bayesian model for dynamic causal modelling of group studies—and thereby limit the effect of outliers using empirical priors. One application of this hierarchical modelling—or empirical Bayes—is to finesse local maxima problems by replacing the full priors over parameters with empirical shrinkage priors from the between-subject level. Effectively, the empirical shrinkage priors pull the within-subject estimates towards the global mean (Friston et al., 2015b). This entails optimizing the parameter estimates for each subject iteratively using priors that are informed by all subjects. In other words, the empirical (between-subject) priors replace the original priors and single-subject inversions are repeated until convergence. This scheme ensures that the local maximum closest to the group posterior mean are obtained for each subject, rendering model inversion more robust to local maxima (and related convergence issues). This is the inversion scheme used in the current report to assess reproducibility over different data and models. We also asked how the scheme will perform in the absence of experimental effects; i.e., when modelling null data.

In what follows, we address some elementary but key reproducibility issues using BMR and empirical Bayes; asking whether one would reach the same inferences or conclusions if one analyzed different data (acquired at the same time from the same subjects), used a formally different model of neuronal dynamics or, indeed, elected to use different inversion schemes that would be otherwise identical under linear models.

In brief, we used empirical data from multiple participants in an auditory mismatch negativity study (Garrido et al., 2007b). To generate two independent but equivalent data partitions, we averaged odd-numbered trials and separately averaged even-numbered trials. Within each of these we produced event related potentials for two experimental conditions: standard and deviant. An additional “null” data partition was generated by combining the standard trials from the odd data partition with standard trials from the even data partition. In these data, any differences between evoked responses are solely due to random between-trial effects. In short, we created two equivalent data partitions of deviant and standard event-related potentials (ERPs) from the same subjects and a null data partition. We then modelled these data using two variants of DCM for electrophysiological responses; namely, the classic ERP model (David et al., 2006), based upon three coupled neuronal masses per source and a more recent CMC model (Bastos et al., 2012, 2015), based upon a CMC that is equipped with four populations. The CMC model is becoming popular because it allows people to differentiate between superficial and deep pyramidal cells that are the sources of forward and backward connections, respectively. Finally, we compare and contrast analysis strategies that have been adopted in the previous literature (analysis of the grand average response over subjects) with the current empirical Bayes approach. These alternatives produced 12 analysis streams (i.e., three data sets, times two neuronal models, times two inversion schemes), enabling us to examine reproducibility over data, models and inversions—and any interactions among these factors. For each of the 12 analyses, we used BMR to evaluate the evidence for 16 connectivity models, i.e., alternative combinations of condition-specific changes in connectivity—and then report the posterior estimates under the best model, or in terms of their Bayesian model average.

This article comprises three sections. The first section briefly reviews the data used to assess reproducibility and the issues inherent in the dynamic causal modelling of such data; with a special focus on group studies and the various choices people have to contend with. The second section describes the data and model inversion procedures used to address reproducibility. The final section presents the results of these analyses and provides an evaluation of reproducibility in terms of Bayesian model comparison (at the level of connectivity models) and Bayesian model averaging (at the level of model parameters).

## Materials and Methods

### EEG Data

The data used in the present study were originally acquired and described by Garrido et al. (2007b). Eleven subjects’ data were used (three female, ages 24–35), as in the original study. Each subject gave signed informed consent before the study, which proceeded under local ethical committee guidelines. EEG activity was measured during an auditory “oddball” paradigm, in which subjects heard “standard” (1000 Hz) and “deviant” tones (2000 Hz), occurring 80% (480 trials) and 20% (120 trials) of the time, respectively, in a pseudo-random sequence. The stimuli were presented binaurally via headphones for 15 min every 2 s. The duration of each tone was 70 ms with 5 ms rise and fall times. The subjects were instructed not to move, to keep their eyes closed and to count the deviant tones.

EEG data were recorded with a Biosemi system with 128 scalp electrodes (BioSemi B.V., Amsterdam, NL, USA). Data were sampled at 512 Hz. The data pre-processing was done anew in SPM12 (http://www.fil.ion.ucl.ac.uk/spm/software/spm12/) using the same pipeline as in the original study: the data were referenced to the average of all channels, high-pass filtered above 0.5 Hz (Butterworth fifth order filter), downsampled to 200 Hz, low-pass filtered below 40 Hz (Butterworth fifth order filter) and epoched between −100 and 400 ms relative to the tone presentation. Trials containing artifacts were rejected by thresholding the absolute amplitude at 100 μV. Channels with more than 20% bad trials were flagged and precluded from further analysis. At this stage, the remaining trials were split into two partitions, with odd and even trials averaged separately, ensuring that both experimental conditions were equally represented. The trial numbers were, by construction, the same for both odd and even data partitions: 195–232 trials for the standard condition (mean: 211) and 48–59 trials for the deviant condition (mean: 54). Averaging was performed using robust averaging (Wager et al., 2005) and the resulting ERPs were low-pass filtered, again at 40 Hz, to remove any high frequency content introduced by the averaging procedure. This resulted in two group data partitions of 11 subjects, one for odd and one for even trials. For each data partition the grand average was computed by averaging over subjects.

An additional null data partition was generated in a similar way. In the null data partition, standard trials were the same as in the odd data partition and “deviant” trials were drawn from the standard condition of the even data partition. Hence, all trials contained responses to standard tones and should therefore not show any condition-specific effects. The number of trials per condition was matched across the three data partitions.

### Dynamic Causal Modelling

**Figure 1:**
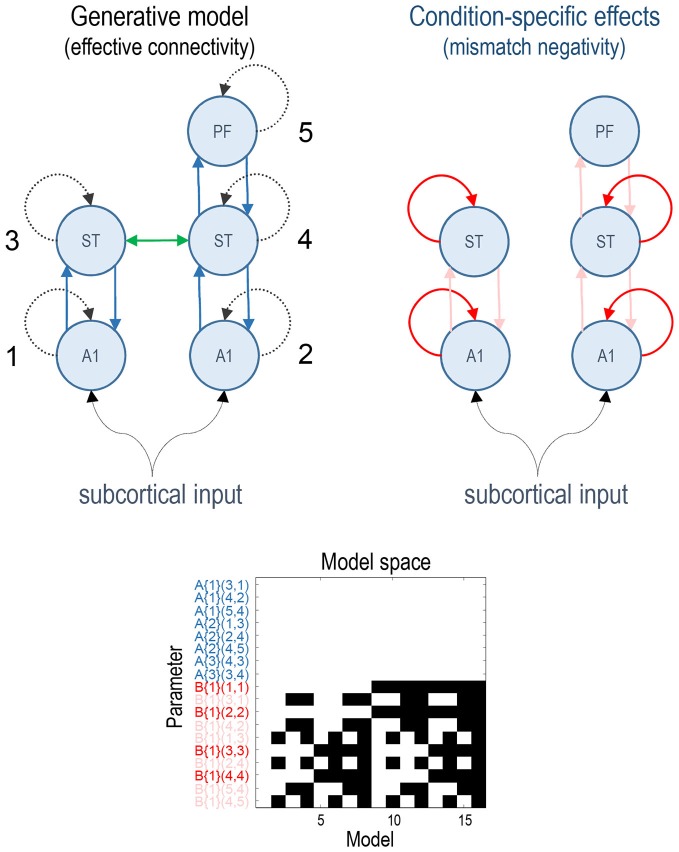
**This figure shows the basic model or network of five (equivalent current dipole) sources used to explain the ERP data.** These constitute primary auditory (A1) sources, higher superior temporal (ST) and prefrontal (PF) sources (on the right). The interesting parameters of this model are the intrinsic and extrinsic coupling or effective connectivity parameters corresponding to the dashed and solid lines, respectively. These are the (random effects) model parameters and are divided into connections that are common to both conditions (A parameters in blue) and those that show condition-specific (i.e., mismatch negativity) effects (B parameters, extrinsic modulatory connections in pink, intrinsic—in red). The green lateral connection between the ST sources was only included in the ERP model. The lower panel shows the model space using a matrix of indicator variables, where white indicates a parameter that is enabled. These variables indicate which parameters constitute each of the 16 models considered, where the parameters are listed as connections among numbered sources (included in the upper right panel). The 16 models correspond to all combinations of models with and without changes in: intrinsic sensory (A1), intrinsic higher (ST), forward and backward connectivity.

Previous studies have established a network of five cortical sources that are sufficient to explain the form of the auditory evoked responses, where differences between standard and deviant stimuli can be accounted for by differences in connectivity both within (intrinsic) and between (extrinsic) sources (Garrido et al., 2007a,b). This network consists of bilateral primary auditory cortex (A1), bilateral superior temporal gyrus (ST) and a prefrontal source at the right inferior frontal gyrus (PF). These three sets of sources comprise three hierarchical processing levels linked by forward connections from A1 to ipsilateral ST and from right ST to right PF and backward connections reciprocating the forward connections. In addition, we also included lateral connections between the superior temporal regions (for the ERP model which allows modelling of lateral connections). This rendered the ERP model identical to that used in the original article (Garrido et al., 2007b). See the upper panels in Figure 1 for a full description.

All of the connectivity models had the same basic architecture and differed only with respect to which connections were allowed to vary to explain the difference between standard and deviant responses. We divided the connectivity model connections into four groups: (i) intrinsic connections of A1 sources (sensory); (ii) intrinsic connections of ST sources (higher); (iii) all forward connections; and (iv) all backward connections. The model space was defined as all possible combinations of these four subsets resulting in 16 (2 × 2 × 2 × 2) connectivity models. The full model allowed for all four subsets of connections to change between standard and oddball conditions. The lower panel of Figure 1 depicts the resulting model space in terms of model parameters that, *a priori*, were allowed to change between conditions. In these DCMs, the synaptic connectivity among neuronal subpopulations is encoded by an A (or weighted adjacency) matrix, while their condition-specific changes are encoded by a matrix termed “B” (see equation 4 in Kiebel et al., 2008). Note that only the B parameters differ among models—it is these parameters that model condition-specific effects; here, the difference between standard and deviant responses.

The analyses were repeated with two types of neuronal model available in the SPM software: the ERP model (Jansen and Rit, 1995; David and Friston, 2003; David et al., 2006) and the Canonical Microcircuit (CMC) model (Bastos et al., 2012, 2015; Moran et al., 2013). In the CMC case, the lateral connections between STs were omitted because they are not supported in this model. The key differences between the ERP and CMC models include a different number of neuronal populations per sources (three vs. four), which effectively splits the pyramidal cell population of the ERP model into superficial and deep pyramidal cell populations in the CMC model. Crucially, condition-specific changes in intrinsic (within-source) connectivity are modelled in a qualitatively different fashion: in the ERP model, the sensitivity of pyramidal cells is modelled directly in terms of postsynaptic excitability or gain (Kiebel et al., 2007). Conversely, in the CMC model (Bastos et al., 2012) condition-specific effects are modelled through changes in self-inhibition of superficial pyramidal cells, such that a *decrease* in self-inhibition produces an increase in excitability or postsynaptic gain. We, therefore, anticipated that condition-specific or mismatch effects would produce opposite changes in intrinsic connectivity in the ERP and CMC models.

### Model Inversion

Model inversion was performed in two ways. First, we fitted the full DCM to each of the group data using the (parametric) empirical Bayesian inversion scheme with empirical (between-subject) shrinkage priors described above (using **spm_dcm_peb_fit.m**). Second, we fitted the full model to the grand averages, as in the conventional approach (using **spm_dcm_fit.m**). The group inversion provided posterior expectations over all model parameters for each subject, while the grand mean inversion estimated the equivalent posterior densities over the parameters of a model generating grand mean responses.

Having inverted the full model using both procedures, BMR was used to estimate the posteriors and model evidence for all reduced models (using **spm_dcm_bmr.m**). For the grand mean analysis this furnished posterior distributions over the 16 models and Bayesian model averages of the connectivity parameters (and condition-specific effects) for the grand mean responses. For the group inversion, group means were estimated (using **spm_dcm_peb.m**) by treating connectivity parameters (and their condition-specific changes) as random effects, while treating all other parameters as subject-specific fixed effects (i.e., parameters pertaining to the stimulus input and spatial parameters such as dipole location and orientation). Subsequent Bayesian model comparison and averaging (using **spm_dcm_peb_bmr.m**) returned posterior distributions over models and densities over group mean parameters. These two inversion (group and grand mean inversion) schemes were repeated for all three (odd, even and null) data partitions under the two (ERP and CMC) neuronal models. For technical and mathematical details please see Friston et al. (2015a,b) and the annotated Matlab routines above (available in the SPM academic software).

## Results

Our objective was to examine reproducibility in terms of inference about condition-specific effects, based upon Bayesian model comparison and averaging. Note that this is not a quantitative test of reproducibility in terms of parameter estimates but a test of reproducibility in terms of models and architectures. In other words, we wanted to assess the reproducibility of inferences about models (to identify functional architectures) and the parameters (synaptic connection strengths and their condition-specific effects) that constitute those architectures. In what follows, we describe the results of Bayesian model comparison and model averaging for the analyses of the grand mean and group data, using odd and even trials.

**Figure 2:**
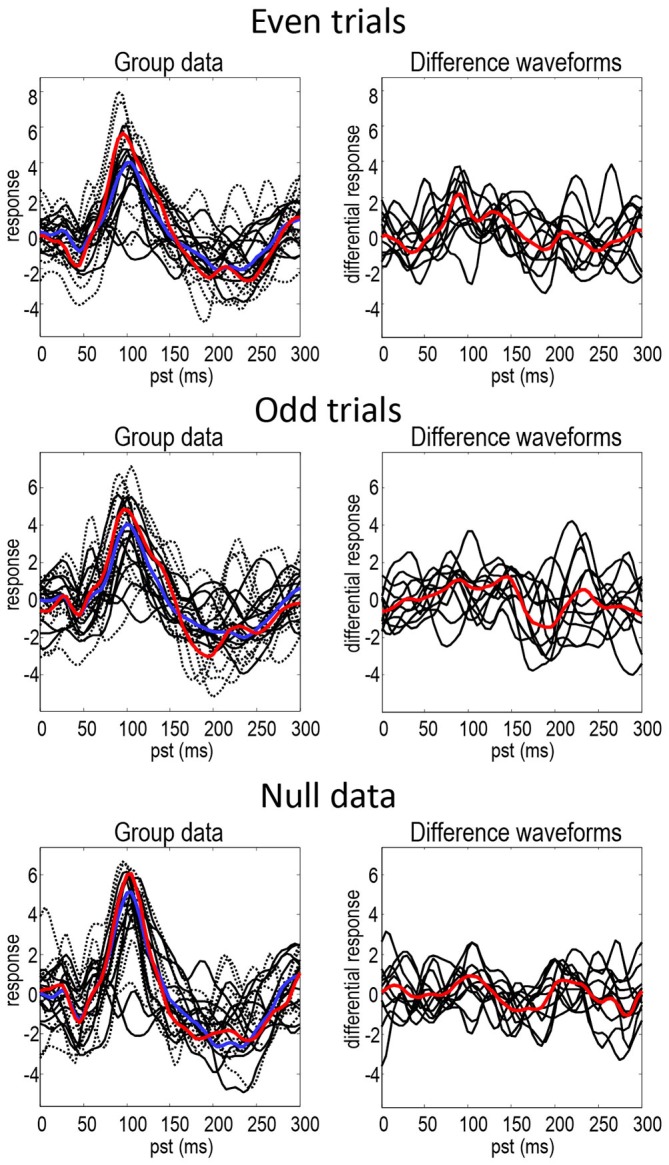
**This figure shows the responses of each subject projected onto the first principal component of the predictive prior covariance matrix, for the standard (full lines—individuals, blue line—mean) and deviant (dotted lines—individuals, red line—mean) conditions on the left—and their differences on the right.** The black lines illustrate the intersubject variability, while the red and blue lines represent the group means.

Figure 2 shows the responses of each subject projected onto the first principal component of the predictive prior sensor covariance matrix. This is the method of dimensionality reduction used in DCM code and is similar to principal component analysis, but based on sensor covariance predicted by the model rather than computed from the data. The standard (full lines—individuals, blue line—mean) and deviant (dotted lines—individuals, red line—mean) conditions are on the left and their differences are on the right. The black lines illustrate the degree of intersubject variability, while the red and blue lines represent the group means. It can be seen that the mismatch negativity (at around 180 ms) is evident in the difference waveforms in both odd and even trials, although possibly more marked in the odd data partition. There is also a hint of an enhanced positivity at around 100 ms. These data show the intersubject variability even in the trial averages—that propagates through to the group means in a nontrivial way. The null data show smaller differences between conditions but when visually inspecting the data the discrepancies between null data and data with real effects are not striking. This indicates that the effects in this dataset are relatively weak and noisy making it especially challenging for DCM (note that splitting the data into odd and even trials reduces the number of trials by a factor of two).

**Figure 3:**
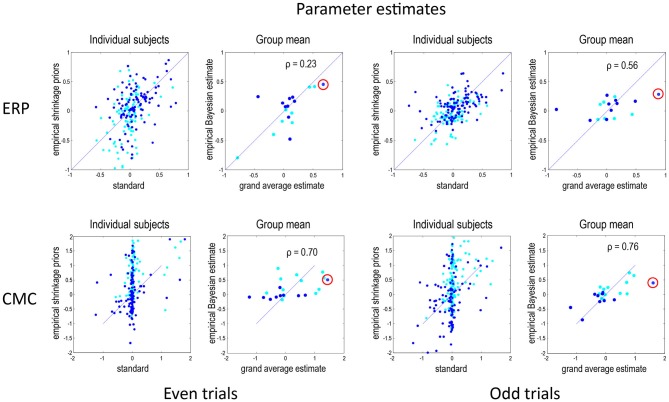
**This figure reports the results of model inversion in terms of the maximum a posteriori (MAP) estimates of connectivity.** These results are shown in the same format for even trials (left panels) and odd trials (right panels) and for the ERP (top row) and CMC (bottom row) models. The dark blue dots correspond to the condition-specific effects (B parameters), while the cyan dots report the common connections (A parameters). The left panels for each data partition show the results following group inversion with empirical (between-subject) shrinkage priors, plotted against the equivalent estimates before repeated application of shrinkage priors, i.e., standard estimation. The right panels show the group mean parameter estimates, following group inversion with empirical shrinkage priors, plotted against the estimates based on the grand mean data. Note that left panels show values for all subjects combined whereas the right panels—for the group mean. In DCM for electrophysiological data, connectivity parameters are log scale parameters. In other words, a parameter of zero corresponds to scaling of *log*(0) = 100%. For connectivity parameters, the scaling is applied to prior expectations (usually of a weak connectivity). For condition-specific effects, a parameter of zero corresponds to no change. The red circles emphasise a condition-specific effect that was identified as the largest in all analyses. This was an increase in the strength of the forward connection to prefrontal source. Pearson correlation coefficients are shown for the B parameters where relevant.

Figure 3 shows the results of model inversion in terms of the maximum a posteriori (MAP) estimates of connectivity. These results are shown in the same format for even trials (left panels) and odd trials (right panels) and for the ERP (top row) and CMC (bottom row) models. The dark blue dots correspond to the condition-specific effects, while the cyan dots report the underlying connection strengths. The upper left panel shows the results following group inversion with empirical (between-subject) shrinkage priors after convergence (four inversions), plotted against the equivalent estimates after the first inversion (which is equivalent to the standard individual inversion). The key thing to note here is that if the Laplace assumption was correct (e.g., as in linear models), these estimates should be identical. In other words, repeating model inversion should not change the estimates. However, it can be seen that there are marked differences due to violations of the Laplace assumption that induce local maxima. This suggests that the initial estimates were, in part, confounded by local maxima. The right panels show the group mean parameter estimates, following group inversion with empirical shrinkage priors, plotted against the equivalent standard individual estimates based on the grand mean data. Although the correlations range between 0.23 and 0.76, it is reassuring to note the same connectivity parameter change (red circles) has been identified as the largest in all four analyses (odd and even in both ERP and CMC models).

Under the CMC model, the correlations between the estimates of the group mean parameters, following empirical Bayes, and the parameter estimates for the grand mean are remarkably similar, and higher than under the ERP model. Interestingly, the subject-specific parameter estimates in the absence of empirical shrinkage priors (after the first inversion) show a rather sparse (heavy tailed) distribution, with a large number of parameters showing very small values and a small number with large values. This sparsity is resolved when using empirical shrinkage priors (after the fourth inversion). One explanation for this is that the prior expectations (of zero) may be a local maximum for many parameters.

**Figure 4:**
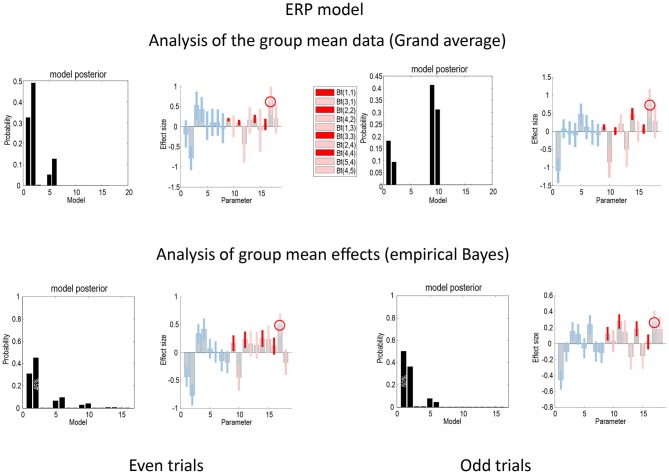
**This figure compares the Bayesian model comparison and averaging results obtained following an analysis of the grand average and empirical Bayesian analysis of the group data, for even (left) and odd (right) trials, using the ERP model.** The key thing to note here is that the profile of model evidences is more consistent following empirical Bayesian inversion; suggesting that models with increases in intrinsic sensory and forward connectivity have the highest evidence. Furthermore, the Bayesian model averages are consistent between the odd and even analyses—and with the analysis of the grand mean. The gray bars correspond to posterior estimates, while the colored bars represent 90% Bayesian confidence intervals: average (A parameters) connections are shown in blue, while connectivity changes (B parameters) are shown in red for intrinsic connections and pink for extrinsic connections. In all four analyses, the largest effect is seen in the forward connection to the prefrontal source (circled). With the exception of two connections in both analyses, the posterior densities over individual connections are internally consistent between odd and even trials.

**Figure 5:**
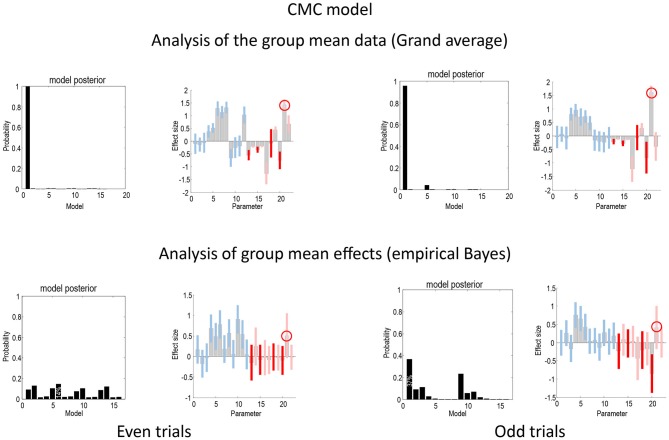
**This figure presents the results of the same analyses presented in Figure 4 but for the CMC model**.

**Figure 6:**
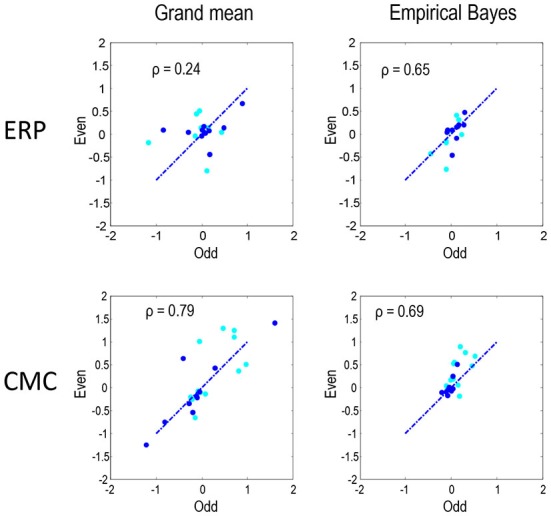
**To quantify the reproducibility of MAP estimates of connectivity shown in Figures 4, 5 these estimates are compared directly as in Figure 3.** Each plot shows the reproducibility of MAP estimates for the odd and even partition under a particular neural model and inversion procedure combination. The dark blue dots correspond to the condition-specific effects (B parameters), while the cyan dots report the common connections (A parameters). Pearson correlation coefficients were computed by pooling both sets of parameters.

**Figure 7:**
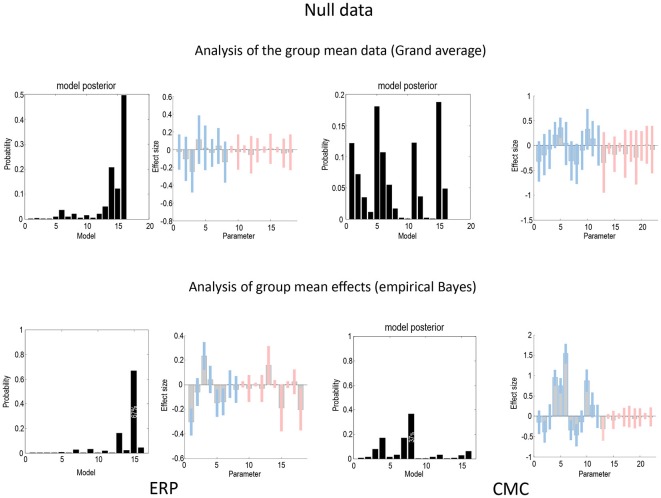
**This figure uses the same format as Figures 4, 5 to report the results of a null model (with no changes in connectivity between odd and even standard trials) for the ERP (left) and CMC (right) neuronal models.** As one would expect, the evidence for a null model (model 16) is clearly non-zero for both the analysis of the grand mean (top row) and empirical Bayesian analysis (bottom row). Furthermore, Bayesian model averaging in both cases shows that the Bayesian credible intervals for parameters showing condition-specific effects include the prior mean of zero. In other words, there is no connection that could be inferred to change with 90% confidence or more. The only exception is a reduction in the last backward connection that nearly reaches 90%.

Figure 4 presents the Bayesian model comparison and averaging results obtained under the ERP model, following an analysis of the grand average and empirical Bayesian analysis of the group data, for even (left) and odd (right) trials. The upper row reports the results of Bayesian model comparison and averaging of the grand mean data. The lower row shows the equivalent results following empirical Bayesian analysis. The model evidences following inversion of the odd and even trials are similar but not consistent. For the grand mean inversion in the even trials, there is clear evidence for increases in intrinsic sensory and forward connectivity, while, for the odd trials, the mismatch negativity related increases have been attributed to the higher intrinsic and forward connectivity. The key thing to note here is that the profile of model evidences becomes more consistent between odd and even trials following empirical Bayesian inversion; suggesting that models with increases in intrinsic sensory and forward connectivity have the highest evidence (see also Figure 5,6,7). In terms of parameters, the Bayesian model averages are consistent between the odd and even analyses—and with the analysis of the grand mean: in all four analyses, the most marked effect is seen in the forward connection to the prefrontal source. Careful inspection of the Bayesian confidence intervals shows that, with the exception of two connections in both analyses, the posterior densities over individual connections are internally consistent between odd and even trials. Crucially, in every analysis, the intrinsic connectivity (red confidence intervals) has increased for deviant trials in all four sources. Furthermore, in all four analyses, the increase of intrinsic connectivity in the fourth source is much less marked than the remaining sources.

Figure 5 shows the same analyses as in Figure 4 for the CMC model. In terms of the changes in connectivity, there is a remarkably consistent profile, in relation to the equivalent results under the ERP model. Again, we see the largest experimental effect is an increase in the forward connection from the superior temporal source to the inferior frontal source—in all four analyses (circled in the figure). Furthermore, in all four sources the intrinsic connectivity shows a disinhibition, in line with the differences in the way intrinsic connectivity is mediated in the two neuronal models. In terms of model comparison, we again see that the empirical Bayesian analysis is more conservative than the Bayesian model comparison based upon the grand averages. In this example, grand average analysis appears to be overconfident and prefers the full model (model 1) for the two data partitions. In contrast, the empirical Bayesian model comparison is much more agnostic about the best model. This is particularly marked for the even trial data partition, where all models have a relatively low probability. For the odd trial data, the preferred model is the same as the (full model) selected using the ERP model.

Figure 6 compares the parameter estimates for the odd and even partitions directly for the two neural models and two inversion procedures. The Pearson correlations coefficients that we present here quantify reproducibility and ranged between 0.24 for the ERP model and grand mean combination and 0.79 for CMC grand mean, with empirical Bayes showing similar values for both ERP and CMC: 0.65 and 0.69 respectively. In summary, the parameter estimates are fairly consistent when using the two neuronal models, the two data partitions, and the two inversion schemes. In terms of model comparison, the empirical Bayesian estimates of model evidence are less subject to overconfidence. For the ERP model they were consistent over independent data; however, for the CMC model the model evidences were less informative.

**Figure 8:**
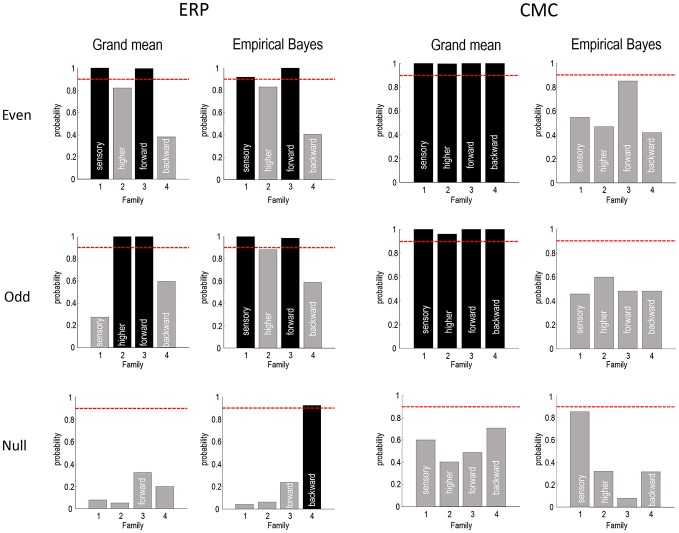
**This figure summarizes the Bayesian model comparisons of Figures 4, 5, 7 in terms of four independent Bayesian family comparisons that assess the evidence for changes in intrinsic sensory and higher (within A1 and ST, respectively) and extrinsic (forward and backward) connectivity.** The posterior probability of the models that include these effects are shown as a bar graph. Note that each bar pertains to a separate hypothesis and therefore the probabilities do not sum to 1. This form of family-wise Bayesian model comparison highlights the consistency following empirical Bayesian group inversion (right panels), that is less complete under the analysis of the grand mean (left panels). However, the only inconsistent result is seen with analysis of the grand mean data from odd trials, in which the changes in intrinsic sensory connectivity have been replaced by changes in intrinsic higher connectivity. The lower row reports the same results using a null analysis comparing odd and even standard trials. The red lines correspond to a 90% posterior probability of each family of models.

Figure 7 uses the same format as Figures 4, 5 to show the results of a null analysis (with standard trials drawn from the odd and even partitions of data). As one would expect, we found highest evidence for a null model (model 16—with no changes in connectivity) for the analysis of the grand mean (top row) and a nearly null model (model 15), for the empirical Bayesian analysis (bottom row) of the ERP model. Furthermore, Bayesian model averaging in both cases shows that the Bayesian credible intervals for parameters showing condition-specific effects include the prior mean of zero. In other words, there is no connection that could be inferred to change with 90% confidence or more. The only exception is a reduction in the last (backward) connection that nearly reaches 90% confidence for the CMC case—and explains why the backward family (see Figure 8) attains 90% confidence. This is not unexpected given the large number of parameters across all the analysis streams and the probabilistic nature of Bayesian inference. The clear difference between the null case and the two partitions with real experimental effects is a pleasing result, which suggests that Bayesian inference precludes experimental effects that are not present, even when the data are suggestive of condition-specific effects on visual inspection (see Figure 2).

Figure 8 summarizes the Bayesian model comparison of the previous figures by grouping the models into four independent “families”, according to whether they include changes in sensory intrinsic, higher intrinsic, forward or backward connectivity. The posterior probabilities of these families are shown as a bar graph, where each bar pertains to a separate hypothesis test (whether a change in the respective connection type is present). The red lines correspond to a 90% posterior probability of each family of models. For the ERP model, this form of family-wise Bayesian model comparison highlights the consistency following empirical Bayesian group inversion (right panels), that is less complete under the analysis of the grand mean (left panels). However, the only inconsistent result (for ERP models) is seen with analysis of the grand mean data from odd trials, in which the changes in intrinsic sensory connectivity have been replaced by changes in intrinsic higher connectivity. Otherwise, we would reach the same conclusion for all analyses of data with real experimental effects. The results for the CMC model are less compelling, showing the overconfident preference for all connectivity changes when inverting the grand average—and uninformative model comparison when using empirical Bayes. Having said this, the ordinal profile of model evidences for the even trials under the CMC model is exactly the same as that obtained under the ERP model, for both the grand mean average and empirical Bayesian analyses. Furthermore, in all analyses the evidence for changes in backward connectivity suggests we can dismiss this as a plausible explanation for oddball responses.

The lower rows report the same results using a null analysis comparing odd and even standard trials. With one exception, all 16 model comparisons suggest negative or weak evidence in favor of any experimental effect. Indeed, the majority of comparisons suggest there is evidence against an experimental effect, with a posterior probability of connectivity changes of less than 50%. With 16 comparisons, one might expect one comparison to exceed a 95% confidence threshold, which is what we observe here. However, note that model comparison is a probabilistic statement. In other words, there is no notion of a false positive because there is still a 5% probability that the null model is the best explanation for the data.

To see whether increasing the amount of data would boost the efficiency of empirical Bayesian inversion, we applied this procedure to the full dataset with odd and even trials combined. In this case, the effect of sensory intrinsic connections reached a posterior probability close to unity—with the other effects staying uninformative.

## Discussion

In this technical note, we have compared conventional (grand mean averaging) analyses of group DCM studies with hierarchical or empirical Bayesian model averaging. Our particular focus was on reproducibility when analysing independent data, under formally distinct models. The main conclusions of this comparative evaluation are as follows: first, the parameter estimates based upon grand mean data are similar to the Bayesian model averages following an empirical (hierarchical) Bayesian analysis—in which condition-specific effects (changes in connectivity) are treated as random effects at the second (between subject) level. Second, Bayesian model comparison based upon the variational free energy as a proxy for log model evidence is more consistent following empirical Bayesian analysis, relative to analysis of the grand mean. Third, empirical Bayesian parameter estimates are consistent over different data partitions and neuronal models.

The sort of reproducibility we have focused on pertains to inferences about functional architectures and condition-specific effects. For the ERP model, a standard way of reporting these results could be something like the following:

“Using Bayesian model comparison, we assessed the evidence for changes in intrinsic sensory, intrinsic higher, forward and backward connectivity, induced by the deviant condition (and underlying the mismatch negativity). The evidence for models with and without each of these changes was assessed using Bayesian family comparison—based upon the posterior density over changes in connectivity at the group level. These model comparisons provided clear (greater than 90% confidence) evidence for increases in intrinsic sensory and forward connections, with relatively little evidence for changes in backwards connectivity. The evidence for changes in intrinsic connectivity at the higher level was positive but not strong (between 80–90%). These conclusions were consistent with the Bayesian model averages of each parameter (accounting for uncertainty over models), with the largest effect (greater than 20% increase) in the forward connection to the prefrontal source. This increase was accompanied by smaller but bilateral increases in the intrinsic connectivity or excitability of the sensory sources, and unilaterally on the right superior temporal source.”

One would then discuss these results in relation to their implications for particular theories of cognitive processing; for example, predictive coding:

“These results are consistent with a predictive coding formulation of the mismatch negativity in terms of oddball or violation responses. In brief, one can associate the increase in forward connectivity in terms of an increase in bottom-up prediction error signalling, when stimuli elude top-down prediction. Conversely, increases in sensory intrinsic connectivity may reflect the exogenous attentional effects of an unpredicted stimulus, which—in predictive coding—correspond to an increase in the precision or gain of superficial pyramidal cells reporting prediction errors.”

Crucially, these conclusions would be exactly the same for the empirical Bayesian analyses of the group data, irrespective of whether we had analyzed the odd or even data partition (or the grand mean of even trials). Furthermore, we would not have been able to articulate these conclusions had we analyzed the null data (where experimental effects were deliberately destroyed).

For the CMC model the Bayesian model comparison (based on the empirical Bayesian analysis) was less informative and would not justify the above conclusions even though the profile of parameter estimates was very similar. This probably reflects the fact that the CMC model has a greater complexity (i.e., has more parameters) and a more biologically realistic form of intrinsic connectivity gain control. This means we would probably require more data to reach the same conclusions. Indeed, if we pooled the odd and even trials, the Bayesian model comparison would then partially support the above conclusion with regards to modulation of sensory intrinsic connections.

Although these results are reassuring, they should only be taken as proof of principle that empirical Bayesian modelling, with dynamic causal models, can yield reproducible results. Clearly, a better sense of the reproducibility of DCM will rest upon future studies, using different models and data features. The example used here was deliberately challenging for DCM. We chose to use data that will be made available as an exemplar (multisubject) EEG dataset (and that have been previously reported). However, these data were acquired from a relatively small number of subjects. Furthermore, the number of trials per condition was small after splitting the trials into odd and even subsets. The ensuing lack of statistical efficiency may explain why the analysis of the null data did not point clearly to a null model (with no condition-specific effects). In this context, there is a fundamental difference between a failure to identify a model that is more likely than any competing models and a confident assertion that the null model is the most likely. The profile of model evidences in Figure 7 speaks to a persistent uncertainty about the most likely model—and would normally suggest more (or better) data are required to clearly establish the null (or any other) model as the most likely.

It is pleasing to note that similar parameter estimates could be obtained following the use of a standard (ERP) model and a CMC model. This is a non-trivial result because the forms of the two models are qualitatively distinct; especially in terms of how changes in intrinsic connectivity or gain are modelled. The CMC model is (arguably) more biologically plausible, because it explicitly models changes in excitability through changes in inhibitory self-connectivity. This necessarily involves (implicit) inhibitory interneurons, which are thought to be crucial in establishing (synchronous) gain and nuancing cortical gain control in terms of excitation-inhibition balance.

Finally, we reiterate that our examination of reproducibility is restricted, in the sense that we have only considered a small number of random effects in a specific dataset and paradigm. By analysing odd and even trials, we have effectively precluded session-to-session sources of variability, which could be an important determinant of reproducibility. From the perspective of models, both the ERP and CMC models are based upon the same mathematical form (they are both weakly nonlinear synaptic convolution models). We anticipate that there will be many more studies along these lines; expanding the repertoire of models that are applied to data and indeed, the nature of the data themselves. Indeed, there are current initiatives in which similar analyses are being applied to multisubject fMRI data. The analysis presented in the present article was based on evoked responses, but a similar procedure can be applied to resting data, which are usually epoched into arbitrary fixed-length segments prior to spectral analysis. These segments can be partitioned into odd and even subsets and subjected to the same analyses described in this article. Assessing the reproducibility of dynamic causal modelling may become increasingly important when the (connectivity for synaptic) parameters of these models are used for diagnosis and prognosis in a clinical setting.

## Author Contributions

MG collected the data. KF, VL and PZ developed the analysis methodology. VL performed the analysis. KF and VL wrote the article.

## Conflict of Interest Statement

The authors declare that the research was conducted in the absence of any commercial or financial relationships that could be construed as a potential conflict of interest.
